# Neuroprotective Role of Cyclic AMP Signaling in Dopaminergic Degeneration Induced by a Parkinson’s Disease Toxin, Rotenone

**DOI:** 10.3390/neurosci6010024

**Published:** 2025-03-11

**Authors:** Sazan Ismael, Sarah Baitamouni, Daewoo Lee

**Affiliations:** 1 Neuroscience Program, Department of Biological Sciences, Ohio University, Athens, OH 45701, USA; si196210@ohio.edu (S.I.); sb185215@ohio.edu (S.B.); 2 Department of Biology, Faculty of Science and Health, Koya University, Koya KOY45, Kurdistan Region-F.R., Iraq

**Keywords:** DREADD, *Drosophila*, primary neuronal culture, rotenone, PKA-C1

## Abstract

Parkinson’s disease (PD) is a progressive neurodegenerative disorder characterized by the selective loss of dopaminergic (DA) neurons in the midbrain. While dopamine precursor levodopa and D2 receptor agonists are commonly used to alleviate PD symptoms, these treatments do not halt or reverse disease progression. Thus, developing effective neuroprotective strategies remains a critical goal. In this study, we explored neuroprotective mechanisms in a *Drosophila* primary neuronal culture model of PD, created by administering the environmental toxin rotenone. Using the chemogenetic DREADD (designer receptors exclusively activated by designer drugs) system, we selectively activated cAMP signaling in DA neurons within the rotenone-induced model. Our results demonstrate that increasing cAMP signaling via Gs-coupled DREADD (rM3Ds) is protective against DA neurodegeneration. Furthermore, overexpression of the catalytic PKA-C1 subunit fully rescued DA neurons from rotenone-induced degeneration, with this effect restricted to DA neurons where PKA-C1 was specifically overexpressed. These findings reveal that cAMP-PKA signaling activation is neuroprotective in DA neurons against rotenone-induced degeneration, offering promising insights for developing targeted therapeutic strategies to slow or prevent PD pathology progression.

## 1. Introduction

Parkinson’s disease (PD), the second most prevalent neurodegenerative disorder, is caused by the loss of dopaminergic (DA) neurons in the substantia nigra pars compacta (SNpc) of the midbrain [1,2,3]. Currently, dopamine precursor L-DOPA is being used as a gold standard drug to relieve PD symptoms, but it does not slow or prevent DA neurodegeneration [4,5]. Furthermore, administration of L-DOPA for 5 years or longer causes uncontrollable involuntary movements, called L-DOPA-induced dyskinesia (LID) [6]. Therefore, development of new neuroprotective strategies is in urgent demand to stop or reverse the progression of PD pathology.

Interestingly, dysregulation of the cyclic AMP-PKA signaling pathway has been observed in both postmortem PD brain tissue and PD models [7]. Furthermore, activation of this pathway has been shown to exert neuroprotective effects in PD [8,9]. However, its specific neuroprotective role in DA neurons remains unexplored.

The fruit fly *Drosophila melanogaster* has become an invaluable model for studying human diseases due to the high conservation of gene sequences and functions among flies, rats, and humans [10]. Specifically for Parkinson’s disease (PD), the *Drosophila* genome contains orthologs for all known PD-related genes except *α-Synuclein* [11,12]. Additionally, toxin-induced PD models have been successfully developed in *Drosophila* [13,14,15,16]. Thus, *Drosophila* serves as an excellent model system for investigating PD pathological mechanisms and developing neuroprotective strategies.

By using the *Drosophila* primary neuronal culture as a cellular model of PD, we examined the neuroprotective effect of the cyclic AMP signaling pathway in DA neurons. In this study, a PD toxin, rotenone, has been used because epidemiological studies show exposure to rotenone is associated with increased prevalence of PD among people in rural regions [17]. Moreover, rotenone causes nigral DA neurodegeneration, motor impairments, and histopathological features of PD, such as formation of Lewy bodies [18,19,20,21]. We also used a chemogenetic approach with designer receptors exclusively activated by designer drugs (DREADDs) [22] that can be used to change intracellular cAMP levels. Using *Drosophila* neuronal culture and DREADD, we provide evidence in support of the neuroprotective role of the cAMP signaling pathway in rotenone-induced DA neurodegeneration.

## 2. Materials and Methods

Fly strains: Flies were maintained on standard cornmeal/agar medium at 25 °C (a 12 h light/dark cycle). Wild-type flies (cantonized white-eye stock, *w1118*) were obtained from the Bloomington Drosophila Stock Center (BDSC) and designated as wild type. For DREADD experiments, we used the following lines: UAS-rM3BCs [22], UAS-hM4Di [22], and TH-Gal4 [23]. Additionally, the UAS-PKA-C1.flag line for PKA C1 overexpression was obtained from BDSC. A standard UAS × Gal4 system was used [24]. A dopaminergic driver, TH-Gal4, was crossed with a fly UAS line carrying a DREADD transgene or PKA-C1 lines to selectively manipulate cAMP levels in dopaminergic neurons.

*Drosophila* primary neuronal culture: *Drosophila* neuronal cultures were prepared as previously described [25,26,27]. Embryos were collected by putting adult flies in fly bottles capped with agar plates. After removing the egg chorion with a 50% bleach solution for 4 min, mid-gastrula-stage embryos were picked up under a stereomicroscope in a laminar-flow hood. Neuroblast cells were harvested from the embryos and plated onto round glass coverslips (Bellco, uncoated). Cells from 2 embryos were plated per coverslip. The culture dish containing coverslips was maintained in an incubator at 24–25 °C with 5% CO_2_ for up to 14 days in vitro (DIV). To ensure the culture remained healthy, half of the medium was replaced every 5 days. The culture medium (DDM1) used in this study was prepared according to a previously described protocol [28].

Pharmacological treatments: Rotenone (Sigma) and clozapine N-oxide (CNO) (Tocris) were added to *Drosophila* neuronal culture at 3 DIV. They were dissolved in DMSO. Stocks were stored at −20 °C.

Immunofluorescence assay (IFA): As previously described [25,28], neurons were fixed with 4% paraformaldehyde, blocked, and permeabilized using PBS with Triton X-100 and goat serum. Primary antibodies (anti-TH or anti-GFP) were applied overnight at 4 °C, followed by secondary antibodies (FITC or TRITC) and DAPI for nuclear staining. After washes, coverslips were mounted and imaged with a fluorescence microscope (Olympus IX71) and Spot CCD camera [28].

Quantification of TH(+) DA neurons: To quantify the total number of cells, images of anti-TH and DAPI signals were captured from 5–10 random areas on each coverslip. Anti-TH(+) cells were manually counted during image acquisition, while DAPI signals were analyzed using ImageJ software (1.52p). Total DAPI(+) cells were quantified as previously reported [28].

Statistical analysis: An unpaired Student’s *t*-test was used for group comparisons. Data are presented as mean ± SEM. Significance thresholds are indicated as * *p* < 0.05, ** *p* < 0.01, and *** *p* < 0.001. Each experiment was independently repeated at least three times.

## 3. Results

### 3.1. Rotenone Selectively Degenerates Drosophila Dopaminergic Neurons in Culture

**Figure 1:**
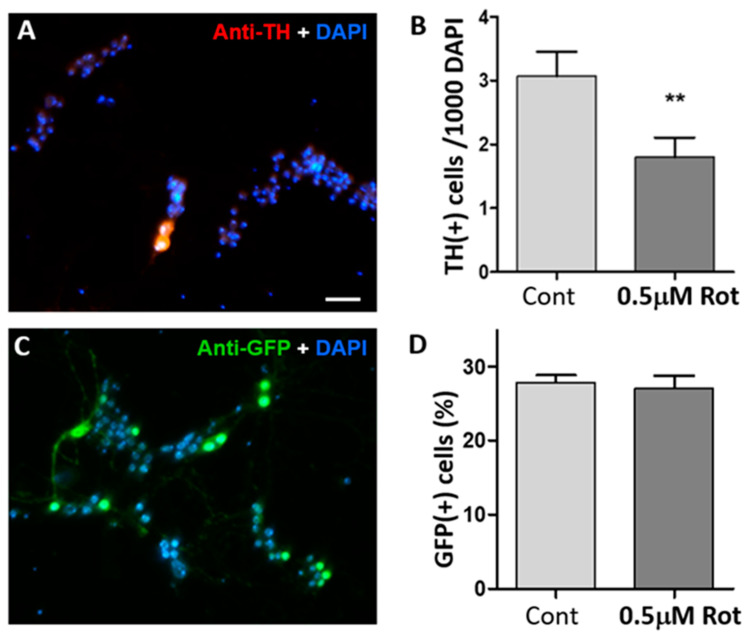
Rotenone selectively degenerates *Drosophila* dopaminergic neurons. (**A**) A representative image of 9 days in vitro (DIV) fly neuronal culture (scale bar, 20 µm). The culture was prepared from wild-type fly embryos. The culture cells were treated with 0.5 µM rotenone at 3 DIV. Cultures were stained with an antibody to tyrosine hydroxylase (TH; red) and DAPI (blue) at 9 DIV. DAPI was used to stain nuclei, enabling the counting of all cells within the field of view. TH(+) neurons were normalized to DAPI. (**B**) Rotenone treatment (0.5 µM) reduces the number of TH(+) neurons in *Drosophila* neuronal culture compared to control (DMSO only). Student *t*-test, ** *p* < 0.01. Number of images analyzed: DMSO (66), Rot (62). All images are from 3 independent experiments. (**C**) Primary neuronal cultures were prepared from flies expressing GFP in cholinergic neurons and treated with 0.5 µM rotenone. Cultured neurons were stained with anti-GFP antibody and DAPI at 9 DIV. GFP(+) neurons were normalized to DAPI. (**D**) Bar graph shows no difference between control and rotenone-treated groups. Number of images: DMSO (68), Rot (39). All images are from 3 independent experiments.

To test whether rotenone degenerates DA neurons, we prepared primary neuronal cultures from wild-type fly embryos. Cultured neurons were observed under a microscope at 3 days in vitro (DIV) to choose the culture coverslips that had a proper density of healthy neurons. The cultures were randomly divided into two groups; cultures were treated with either 0.5 µM rotenone or with DMSO as vehicle control. At 9 DIV, cultures were stained with an anti-TH antibody and DAPI for quantification. The number of DA neurons were quantified by counting TH(+) signals per 1000 DAPI(+) cells, as mentioned in the methods section. Rotenone treatment in *Drosophila* primary culture decreases the DA neuron number significantly (Figure 1). Thus, this culture system mimics a cellular hallmark of PD, validating *Drosophila* primary neuronal culture as a cellular model of PD.

Next, we examined whether rotenone is toxic to other neuronal types. Cholinergic neurons were quantified because they are the main neuronal type in the primary neuronal culture [27]. By using a Cha-Gal4 x UAS-GFP cross, we were able to express GFP selectively in cholinergic neurons (Figure 1C). The number of cholinergic neurons at 9 DIV was not decreased by 0.5 µM rotenone (Figure 1D). The results show that rotenone exposure causes degeneration of DA neurons but is not toxic to other neuronal types, especially cholinergic neurons.

### 3.2. DREADD Activation Modulates Sensitivity of DA Neurons to Rotenone

Designer receptors exclusively activated by designer drugs (DREADDs) are a class of genetically engineered protein receptors, used for chemogenetics approaches, which are selectively activated by certain ligands (e.g., clozapine-N-oxide). DREADDs are engineered muscarinic G protein-coupled receptors (GPCRs) that lack affinity for acetylcholine but respond to the biologically inert compound clozapine-N-oxide (CNO) [22,29]. Activation of Gαs-coupled receptors (e.g., rM3BCs) by CNO increases cAMP levels in a dose-dependent manner, whereas stimulation of Gαi-coupled receptors (e.g., hM4Di) with CNO reduces cAMP levels [22].

Before testing GPCRs (e.g., rM3BDs) activated by DREADs, we wanted to determine whether CNO is toxic to *Drosophila* primary neurons. Wild-type neuronal cultures were treated with different concentrations (i.e., 1 μM, 5 μM) of CNO at 3 DIV, and then stained with anti-TH antibody at 9 DIV. There were three treatment groups: vehicle control (DMSO only), 1 μM or 5 μM CNO. Compared to control, 1 μM CNO did not reduce the number of DA neurons, while 5 μM CNO reduced the DA neuronal number (Table 1). These results showed that 1 μM CNO is not toxic to *Drosophila* primary cultured neurons.

**Figure 2:**
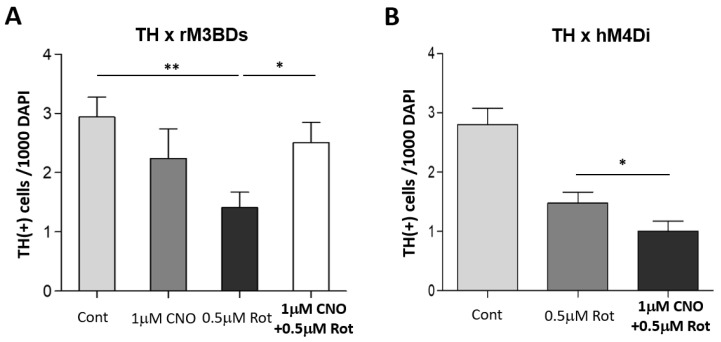
Activation of Gαs and Gαi differently modulate the sensitivity of DA neurons to rotenone. (**A**) Activation of the rM3BD receptor in dopaminergic (DA) neurons protects against rotenone toxicity in *Drosophila* neuronal cultures. Primary cultures were derived from a cross between UAS-rM3BDs and TH-Gal4 flies. At 3 days in vitro (DIV), cultures were treated with rotenone alone or with 1 μM or 5 μM CNO. At 9 DIV, cells were stained with anti-TH antibody, and TH(+) neurons were quantified. Rotenone treatment reduced the number of TH(+) neurons, while activation of rM3BDs with 1 μM CNO rescued the toxic effect. Number of images: DMSO (77), 1 μM CNO (36), 0.5 μM Rot (85), 1 μM CNO + 0.5 μM Rot (99). Data are from 4 independent experiments. (**B**) Activation of the hM4Di receptor in DA neurons exacerbates rotenone toxicity in neuronal cultures. Cultures were derived from the F1 progeny of a cross between UAS-hM4Di and TH-Gal4 flies. At 3 DIV, cultures were treated with rotenone alone or with 1 μM CNO. At 9 DIV, cells were stained with anti-TH antibody and quantified. Number of images: DMSO (105), 0.5 μM Rot (70), 0.5 μM Rot + 1 μM CNO (71). Data are from 5 independent experiments. Statistical significance was determined using Student’s *t*-test, * *p* < 0.05, ** *p* < 0.01.

In the subsequent experiments, we expressed the Gαs-coupled rM3BD receptor in dopaminergic (DA) neurons to investigate the neuroprotective role of cAMP. Neuronal cultures were derived from a cross between UAS-rM3BDs and TH-Gal4 lines and treated with either rotenone alone or rotenone combined with CNO. Activation of the rM3BDs receptor was achieved by applying 1 μM CNO. Cells were stained at 9 DIV with an anti-TH antibody and quantified. Concomitant treatment with 1 μM CNO almost fully rescued the rotenone-induced degeneration of DA neurons at 9 DIV (Figure 2A).

To verify effects of Gαi-coupled hM4Di on rotenone toxicity, primary neuronal cultures were used for immunocytochemistry. We expressed Gαi-coupled hM4Di receptors in cultured DA neurons. Cultures were prepared from a cross between UAS-hM4Di and TH-Gal4. At 3 DIV, cultures were treated with rotenone with or without CNO. Activation of hM4Di receptors was achieved by applying 1 μM CNO. DA neurons were stained at 9 DIV with an anti-TH antibody and quantified. Concomitant treatment of rotenone with 1 μM CNO worsened rotenone-induced degeneration of DA neurons at 9 DIV (Figure 2B), further showing that lower levels of intracellular cAMP are associated with greater susceptibility to rotenone.

### 3.3. PKA-C1 Overexpression Protects Against DA Neurodegeneration

Given that PKA is the primary downstream target of cAMP in *Drosophila*, we investigated its potential neuroprotective role against rotenone toxicity. PKA comprises two subunits: the cAMP-binding regulatory subunit (PKA-R), which dissociates from the catalytic subunit (PKA-C) upon cAMP binding, allowing PKA-C to phosphorylate its substrates [30]. Among the three catalytic subunits of PKA (PKA-C1, C2, and C3), PKA-C1 has been the most extensively studied in *Drosophila* [31]. To activate PKA constitutively in dopaminergic (DA) neurons, we overexpressed PKA-C1 by crossing UAS-PKA-C1 with TH-Gal4.

**Figure 3:**
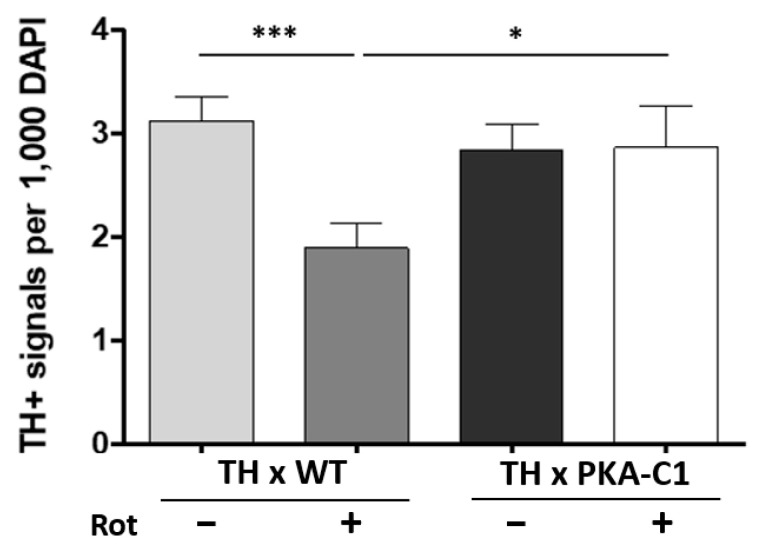
Overexpression of the catalytic subunit C1 of PKA in DA neurons protects against rotenone toxicity in *Drosophila* cell culture. Cell cultures were prepared from F1 progeny of a cross between UAS-PKA-C1 and TH-Gal4 flies (TH x PKA-C1) and a cross between WT and TH-Gal4 flies (TH x WT). At 3 days in vitro (DIV), cultures were treated with either 0.5 μM rotenone or DMSO. At 9 DIV, neurons were stained with anti-TH antibody, and TH(+) neurons were quantified. In the TH x WT cultures, rotenone treatment reduced the number of TH(+) neurons, while no significant change in TH(+) neurons was observed in the TH x PKA-C1 line following rotenone exposure. Number of images: TH x WT (control) (97), TH x WT (Rot) (95), TH x PKA-C1 (control) (64), TH x PKA-C1 (Rot) (40). Data are from 3 independent experiments. Statistical significance was determined using Student’s *t*-test, * *p* < 0.05, *** *p* < 0.001.

Cultured DA neurons expressing constitutively active PKA-C1 were protected from degeneration induced by rotenone (Figure 3). The number of DA neurons in PKA-C1 cultures after rotenone treatment was not different when comparing TH x WT and TH x PKA-C1 cultures without rotenone, demonstrating complete rescue by expressing PKA-C1. These data further confirm the neuroprotective role of cAMP-PKA signaling against rotenone.

## 4. Discussion

We studied neuroprotective mechanisms against DA degeneration induced by an environmental PD toxin, rotenone, using a *Drosophila* primary neuronal culture. Our results show that rotenone damages DA neurons, and this damage is specific to DA neurons, as rotenone treatment did not affect cholinergic neurons in the primary culture system. Using the chemogenetic tool with DREADDs, we also showed that selective activation of cAMP signaling in DA neurons can be neuroprotective in the rotenone-induced PD model. Our study confirms that *Drosophila* primary neuronal culture is an excellent model system not only to study pathological mechanisms of PD, but also to develop neuroprotective strategies.

In this study, we have shown that modulation of cAMP signaling has striking impacts on neuroprotection. Activation of Gαs-coupled receptors increases cAMP levels specifically in DA neurons and rescues the neuronal loss caused by rotenone in a *Drosophila* cell culture model. Conversely, activation of Gαi-coupled receptors reduces cAMP levels and exacerbates dopaminergic (DA) neurodegeneration. Our experiments modulating the cAMP pathway in Parkinson’s disease (PD)-like models using *Drosophila* neuronal cultures suggest that decreased cAMP levels enhance rotenone toxicity, whereas upregulation of this pathway protects DA neurons from rotenone-induced toxicity. Additionally, recent findings from our lab indicate that increasing cAMP levels rescues *Drosophila* larval locomotion deficits caused by rotenone exposure [32]. Together, these findings underscore that activation of the cAMP signaling pathway not only protects DA neurons from rotenone-induced neurodegeneration but also ameliorates PD-like behavioral symptoms.

Neuroprotective mechanisms by cAMP signaling in dopaminergic neurons appear to be dopamine cell-autonomous, as genetic interventions in this study were restricted to DA neurons. The following mechanisms can be possibly activated by increased cAMP signaling. First, PKA is well known to phosphorylate ion channels and receptors. It was shown that the PD-associated LRRK2 mutation is known to reduce the sensitivity to neuronal stimulation via dopamine D1Rs [33]. Thus, synaptic and neuronal function compromised by rotenone can be preserved by increased cAMP. Second, PINK1 is known to enhance neuritic outgrowth via activation of PKA. Thus, it is possible that increased cAMP can restore neuritic morphology and function and thus prevent a ‘dying-back’ mechanism of neurodegeneration [34]. Third, dysregulation of PKA signaling may contribute to Parkinson’s disease (PD) etiology through its effects on mitochondrial function, as mitochondrial dysfunction is observed in various cell-based and animal PD models [7]. In a familial form of PD, cAMP treatment and transient expression of a constitutively active PKA catalytic subunit targeted to the outer mitochondrial membrane (OMM) improved mitochondrial interconnectivity, whereas OMM-targeted PKA inhibitors induced mitochondrial fragmentation preceding cell death [35]. Another study demonstrated that expression of human uncoupling protein 2 (hUCP2), a mitochondrial membrane transport protein, in flies attenuated rotenone-induced mitochondrial fragmentation, likely through elevated intracellular cAMP levels. Conversely, PKA inhibitors abolished mitochondrial integrity improvements conferred by hUCP2 expression [15]. Therefore, our findings suggest that PKA activators or phosphodiesterase (PDE) inhibitors could serve as potential therapeutic agents to preserve dopaminergic neuron lifespan in the substantia nigra of PD patients and slow disease progression. Four, cAMP signaling can mediate neuroprotection through gene expression, as a cAMP-dependent transcription factor CREB is involved in gene expression. Using catecholaminergic cell lines treated with 6-OHDA, Chalovich et al. [36] showed that mRNA levels of neuroprotective factors BDNF and Bcl2 mRNA were reduced following a decrease in PKA signaling, but cAMP treatment rescued the 6-OHDA-induced cell death. A future study using *Drosophila* primary culture with rotenone will aim to examine downstream mechanisms of neuroprotection by cAMP, as *Drosophila* has rich genetic resources to test the possible mechanisms proposed above.

## 5. Conclusions

Our study showed that activation of the cAMP-PKA signaling pathway in dopaminergic neurons is neuroprotective against rotenone-induced neurodegeneration in *Drosophila* primary neuronal culture. This neuroprotection is dopamine cell-autonomous, as it is mediated through PKA activation only in DA neurons. Our results can provide novel insights into the development of new therapeutic treatments against PD pathology progression.

## Figures and Tables

**Table 1:** One μM CNO is not toxic to wild-type *Drosophila* neuronal cultures. Cell cultures were prepared from wild-type flies. At 3 days in vitro (DIV), cultures were treated with no clozapine N-oxide (CNO), 1 μM CNO or 5 μM CNO. At 9 DIV, cells were stained with anti-TH antibody and DAPI for quantification. The number of TH(+) neurons was not different in the 1 μM CNO-treated condition compared to control, but 5 μM reduced TH(+) neurons compared to control. Student *t*-test, ** *p* < 0.01. All data are from 3 independent experiments. N = number of images analyzed.

Treatment	TH(+) Cells/1000 DAPI	N
No CNO	3.09 +/− 0.35	57
1 μM CNO	3.12 +/− 0.47	62
5 μM CNO	1.86 +/− 0.30 **	45

## Data Availability

Data will be made available on request.
